# Mixed Pattern Matching-Based Traffic Abnormal Behavior Recognition

**DOI:** 10.1155/2014/834013

**Published:** 2013-01-27

**Authors:** Jian Wu, Zhiming Cui, Victor S. Sheng, Yujie Shi, Pengpeng Zhao

**Affiliations:** ^1^The Institute of Intelligent Information Processing and Application, Soochow University, Suzhou 215006, China; ^2^Department of Computer Science, University of Central Arkansas, Conway, AR 72035, USA

## Abstract

A motion trajectory is an intuitive representation form in time-space domain for a micromotion behavior of moving target. Trajectory analysis is an important approach to recognize abnormal behaviors of moving targets. Against the complexity of vehicle trajectories, this paper first proposed a trajectory pattern learning method based on dynamic time warping (DTW) and spectral clustering. It introduced the DTW distance to measure the distances between vehicle trajectories and determined the number of clusters automatically by a spectral clustering algorithm based on the distance matrix. Then, it clusters sample data points into different clusters. After the spatial patterns and direction patterns learned from the clusters, a recognition method for detecting vehicle abnormal behaviors based on mixed pattern matching was proposed. The experimental results show that the proposed technical scheme can recognize main types of traffic abnormal behaviors effectively and has good robustness. The real-world application verified its feasibility and the validity.

## 1. Introduction

Trajectory is an intuitive representation form of micromotion behaviors of moving targets in a time-space domain. The trajectories of moving targets with the same motion behavior pattern appear higher similarity and repeatability [1, 2]. Through extracting motion trajectories of traffic vehicles in a certain scene, a time-space distribution of trajectory data set can be obtained. Then, we use pattern classification methods to extract classical motion patterns and further conduct effective research and applications in the fields of traffic scene modeling, traffic behavior recognition and prediction, and abnormal incident monitoring. The analysis of abnormal trajectories is an important method for recognizing abnormal behaviors of moving targets. How to process and analyze real-time motion trajectories and how to recognize traffic abnormal behaviors of moving targets, according to the obtained normal behavior patterns, are important in traffic abnormal behavior detection.

Researchers have conducted in-depth research and have made some progress on recognizing abnormal behaviors of moving vehicles in traffic monitoring. Currently, there are many abnormal behavior recognition methods, and the commonly used methods are decision tree, hidden Markov model, neural network, support vector machine, Bayesian network, and so on. Piciarelli and Foresti [3] used decision tree to segment trajectories of moving vehicles as tree nodes and conducted probability matching to distinguish abnormal incidents. This method depends on initial conditions and is prone to over-fitting. Kamijo et al. [4] proposed a traffic anomaly detection algorithm for traffic intersections. It first detects vehicles under complicated blocked conditions based on a time-space Markov random field. Then, it uses hidden Markov model to distinguish specific collisions, congestions, illegal U-turns, and so forth, according to motion properties of vehicles. Micheloni et al. [5] described any kind of incidents as a combination diagram of temporal and spatial relationships. This diagram is composed of a set of simple incidents, which can be classified by a neural network classifier. Therefore, an incident classifier is a compound of several neural networks. Its classification performance relies on the complementary of these neural networks. If simple classifiers are over-fitting, they will negatively impact the entire classification performance. Piciarelli et al. [6] also used support vector machine to detect abnormal incidents. The computational complexity of this algorithm is very high, and there also exists over-fitting, so it could not realize real-time operations and produce good generalization capability.

As we know, traditional trajectory analyzing methods mainly consider the spatial characteristics of trajectories. Their ability of recognizing abnormal behaviors is weak. They can only recognize simple abnormal behaviors. Hu [7] proposed a method of learning and matching patterns from three aspects (i.e., space, direction, and category) of motion trajectories. It enhances the distinguishing ability for trajectories. However, it still cannot recognize complex abnormal behaviors. Li et al. [8] described motion trajectories using spatial positions, motion speeds, motion directions, and target sizes; conducted joint matching and edge matching based on Bayesian optimization; and then looked up a behavior recognition database to recognize corresponding behaviors of a target according to the matching degree. This method uses a traditional *k*-means algorithm to conduct trajectory clustering, which cannot solve the nonconvex clustering problem.

This paper conducted in-depth research on vehicle motion trajectories and proposed an effective method to recognize traffic abnormal behaviors by trajectory analyzing. We first introduced DTW distance to measure the distance between vehicle trajectories and put forward a pattern learning method of motion trajectories based on DTW and spectral clustering. After we have the distances for every pair of trajectories, we obtain a distance matrix. Then, we use a spectral clustering algorithm to conduct the clustering. Note that the spectral clustering algorithm automatically determines the number of clusters. After obtaining spatial patterns and direction patterns of vehicle trajectories, we conducted traffic abnormal behavior recognition based on mixed pattern matching, which contains the spatial patterns and direction patterns. The experimental results showed that the proposed method recognizes main types of traffic abnormal behaviors effectively. The results also verified the feasibility and validity of our proposed method.

## 2. Trajectory Pattern Learning

### 2.1. A Spectral Clustering Algorithm

In recent years, as a novel clustering method, the spectral clustering algorithm has received widespread attention and become a research focus in the fields of machine learning, pattern recognition, and so on. Spectral clustering just needs a similarity matrix between sample points. The idea of the spectral clustering algorithm comes from a spectrum partitioning theory [9, 10]. Assuming that each sample data is regarded as a vertex set *V* in the graph, the spectral clustering algorithm assigns weights *W* for edges *E* according to the similarities between sample points. Thus, we obtain an undirected weighted graph *G* = (*V*, *E*). Therefore, we can transform a clustering problem into a graph partitioning problem. The spectral clustering algorithm first defines a similarity matrix, which describes the similarity of paired data points according to the given sample data. Then, it calculates the eigenvalues and eigenvectors of the similarity matrix, and selects an appropriate number as the number of clusters to cluster sample points.

The main task of the spectral clustering algorithm is to make full use of eigenvectors or decomposed spectral features, and then, conducts clustering by traditional clustering methods, such as *k*-means, and EM clustering algorithms. Spectral clustering has gained widespread concerns from academic and industrial communities, because spectral clustering does not need any assumption on the global structure of sample data and has the ability to identify the non-convex distribution. It is very suitable for many practical problems.

Initially, Hagen and Kahng [11] applied the spectral clustering algorithm to the field of image segmentation. They used pixels in an image as vertices, determined the weights of edges between pixels in a spectral graph according to the luminance and the spatial position of the pixels, and used a 2-way *N* cut spectral clustering method to conduct graph partitioning iteratively. This method got a satisfactory result. After that, spectral clustering algorithms are applied to the fields of text mining [12, 13] and bioinformatics mining [14, 15].

The steps of the traditional spectral clustering algorithm are as follows: (1) use the Euclidean distance to measure the similarity between sample points and then get a similarity matrix through the transformation of Gaussian kernel; (2) obtain a Laplace matrix by transforming the similarity matrix; (3) calculate the eigenvalues and eigenvectors of the Laplace matrix and sort the eigenvalues according to descending order; (4) build a matrix with eigenvectors in accordance with the first *k* largest eigenvalues; (5) regard each row of the matrix obtained from step (4) as a point in *k*-dimensional space and then use a traditional clustering algorithm to conduct clustering; (6) if the *i*th row of the matrix belongs to cluster *j*, the *i*th corresponding sample is clustered into cluster *j*.

But traditional spectral clustering algorithms need to set up the number of clusters in advance. That is, they cannot determine the number of clusters automatically. To solve this problem, Kong et al. [16] proposed an automatic spectral clustering algorithm based on eigengaps and orthogonal eigenvectors. Compared with traditional spectral clustering algorithms, the improvement of this algorithm is that it first calculates an eigengap sequence, selects the first maxima in the sequence after sorting the eigenvalues of the Laplace matrix in descending order, and then treats the subscript of the first maxima as the number of clusters.

Traditional clustering algorithms, such as the commonly used *k*-means algorithm, require that sample data have the same dimensions. However, the number of tracepoints in each moving vehicle trajectory is obviously different. We cannot directly use the traditional clustering algorithms to cluster vehicle trajectories. Therefore, this paper comes up with a method, which uses DTW to measure the distance between each pair of trajectories and then uses the spectral clustering algorithm to cluster moving vehicle trajectories. However, considering the shortcomings that the traditional spectral clustering algorithm cannot determine the number of clusters automatically, this paper uses the method proposed in the literature [16] to determine the number of clusters automatically and then clusters vehicle trajectories.

### 2.2. Trajectory Similarity Measurement

The similarity of trajectories plays a very important role in clustering motion trajectories. At present, the distance similarity measurement methods in trajectory clustering mainly include the Euclidean distance-based method, the method combining principal component analysis (PCA) with Euclidean distance, the longest common subsequence-based method, and the Hausdorff distance-based method. This paper introduces a DTW distance into the vehicle trajectory space and extends it to measure the similarity from one-dimensional variable-length time-series data to two-dimensional variable-length spatial trajectories.

DTW is a nonlinear reformed technology which combines time reforming with the distance calculation. It is based on the idea of dynamic programming [17] and solves the template matching problem that the lengths of pronunciations are different. It appeared very early in speech recognition. DTW is a flexible pattern-matching algorithm, which can match the patterns with global or local expansion, compression, or deformation [18]. Because the DTW algorithm does not require additional computation in the training stage, it is widely used in isolated word speech recognition. The relationships between the frame numbers of test templates and the ones of reference templates can form a network (i.e., a grid). The cross point of the grid indicates the intersection of a test template and a reference template. The DTW algorithm is to find a best path from a starting point to a goal cross point, which makes comprehensive distortion of all frames corresponding to the cross points on the path minimal.

**Figure 1:**
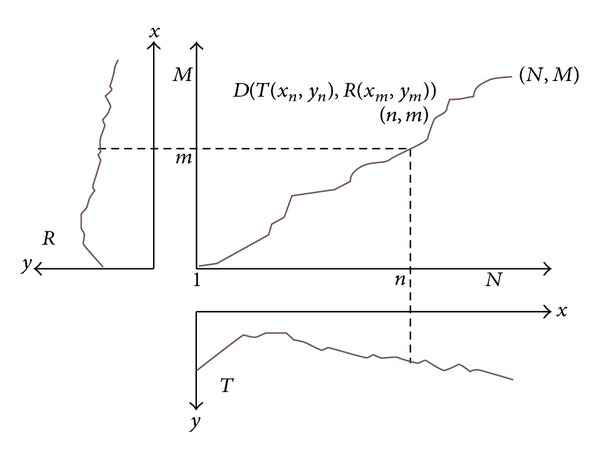
The DTW algorithm for minimum distortion.

The principle of the DTW algorithm in the trajectory similarity measurement is as follows. Assume that trajectory *T* has *N* tracepoints, and trajectory *R* has *M* tracepoints. The number of tracepoints of trajectory *T* is marked as the horizontal coordinate, and the number of tracepoints of trajectory *R* is marked as the vertical coordinate, as shown in the principle diagram Figure 1. So the relationships among tracepoint numbers form a grid. Any cross point (*N*, *M*) in the grid indicates the intersection of trajectory *T*(*x*_*n*_, *y*_*n*_) and trajectory *R*(*x*_*m*_, *y*_*m*_), and the intersection point has a distance metric *D*(*T*(*x*_*n*_, *y*_*n*_), *R*(*x*_*m*_, *y*_*m*_)).

The path selection in the search process of the DTW algorithm is not arbitrary. The traveling direction of different vehicles in the same lane is the same. Therefore, the best path between two trajectories starts from the lower left corner and terminates at the upper right corner. Generally, it does not allow the path tilted excessively to the horizontal axis and the vertical axis. In order to prevent the blind search, there is a provision that the path slope of each point is between 1/2 and 2.

### 2.3. Trajectory Spatial-Pattern Learning

This paper uses the spectral clustering algorithm. It determines the number of clusters automatically to cluster trajectory data with higher spatial similarity. Traditional clustering algorithms, such as *k*-means and EM, are all based on a convex spherical sample space. They fall into local optimal solutions when the sample space is not convex. The spectral clustering algorithm can conduct clustering in any shape of the sample space and converge to a global optimal solution. The spectral clustering algorithm calculates eigenvalues and eigenvectors of the affinity matrix and then selects the appropriate features to cluster all sample data points.

For a trajectory set Track = {*L*_1_, *L*_2_,…, *L*_*n*_}, according to automatic spectral clustering, the clustering steps are as follows.

(1) Use the DTW algorithm to calculate the shortest distance between any two trajectories to construct a distance matrix *D*.
(2) Construct a similarity matrix *W* according to *D*.
(3) Calculate a noncanonical Laplacian matrix *K* from the similarity matrix *W* and the matrix *U*,  *U*_*ii*_ = ∑_*j*=1_^*n*^ *W*_*ij*_.
(4) Calculate the matrix eigenvalues of *K*, sort them in descending order, and find the position of the first maximum eigengap, denoted as *k*. *k* is the number of clusters.
(5) Calculate the eigenvectors *v*_1_, *v*_2_,…, *v*_*k*_ corresponding to the first *k* largest eigenvalues, construct a matrix *S* = [*v*_1_, *v*_2_,…, *v*_*k*_], and construct a matrix *Y* by a unitized processing of each row in the matrix *S*, using the formula as follows:
  (1) $$\begin{array}{c} Y_{ij}=\frac{S_{ij}}{{\left(\sum_{j}S_{ij}^{2}\right)}^{1/2}}. \end{array}$$
(6) Regard each row of the matrix as a point in a *k*-dimensional space. Each trajectory sample *L*_*i*_ corresponds to the *i*th row vector of the matrix *Y*. Use a traditional clustering algorithm to conduct clustering in the new space. Thus, the trajectories are divided into *k* clusters.
(7) If the *i*th row of *Y* belongs to the *j*th cluster, then the trajectory *L*_*i*_ is clustered into the *j*th cluster.

### 2.4. Trajectory Direction-Pattern Learning

According to the learning results of the trajectory spatial patterns, we calculate the central trajectories of different clusters. Thereby we extract the direction patterns of different clusters and further determine the motion direction of moving vehicles.

For a trajectory set Track = {*L*_1_, *L*_2_,…, *L*_*n*_}, the obtained clusters by our algorithm are denoted as Δ = {*C*_1_, *C*_2_,…, *C*_*k*_}. The direction-pattern learning procedure of trajectories is as follows.

1. Calculate the central trajectories of different clusters.
  - Calculate the eigenvalues of the Laplacian matrix and determine the number *k* of clusters. Then we get the matrix *Y*.
  - Construct a data structure Cluster_Vector(*i*, *j*),  *i* = 1,2,…, *n*, *j* = 1,2,…, *k*, according to the matrix *Y*. We get the clustering centers Cluster_Center(*j*),  *j* = 1,2,…, *k* by using a traditional clustering method to cluster sample data points.
  - Search the closest row vector to the clustering center Cluster_Center(*j*), using Euclidean distance. The trajectory of the closest row vector represents the central trajectory of each cluster.
2. For each central trajectory *A* = {*v*_*i*_, *i* = 1,2,…, *m*}, which is a sequence of tracepoints *v*_*i*_ = 〈*x*_*i*_, *y*_*i*_〉, where *v*_1_ = 〈*x*_1_, *y*_1_〉 is the starting point, *v*_*m*_ = 〈*x*_*m*_, *y*_*m*_〉 is the ending point and $\vec{v_{1}v_{m}}$ is defined as the direction-pattern of the corresponding cluster.

## 3. Vehicle Abnormal Behavior Analysis

### 3.1. kNN Classification Algorithm

In the field of data mining, classification is an important technology. It can build a classification model from a set of known training samples and then uses it to classify future samples. Currently, commonly used classification methods are decision tree, neural network, kNN, support vector machine, Bayesian network, and so on [19]. This paper uses the kNN classification algorithm to classify trajectories to recognize motion trajectories.

The kNN classification algorithm was proposed by Cover and Hart originally, which is a mature method [20]. The idea of this method is very simple and intuitive. If most adjacent samples of a sample in a feature space belong to one certain category, this sample most likely belongs to this category. In classification decision, the kNN algorithm only considers one or several nearest neighbors to decide the category the sample belongs to. Because of this it can reduce the negative impact of imbalance issues. In addition, because the kNN algorithm makes prediction for a sample only based on its surrounding adjacent samples, it is more appropriate than other methods for the sample set with more intersection and overlapping.

Although kNN reduces the negative impact of imbalance, it does not remove the impact completely. While the samples are imbalance, for example, one classification's sample capacity is very large while other categories' sample capacities are relatively small, it would lead to the following result. When classifying a new sample, if the sample belongs to one category with a small sample capacity, but its nearest neighbors belong to a category with a great sample capacity, it most likely is classified into the category with a great sample capacity. Thus, a classification error occurs. Another disadvantage of kNN is its computing cost, because the distance of the sample to all known samples must be computed before making the prediction for it.

**Figure 2:**
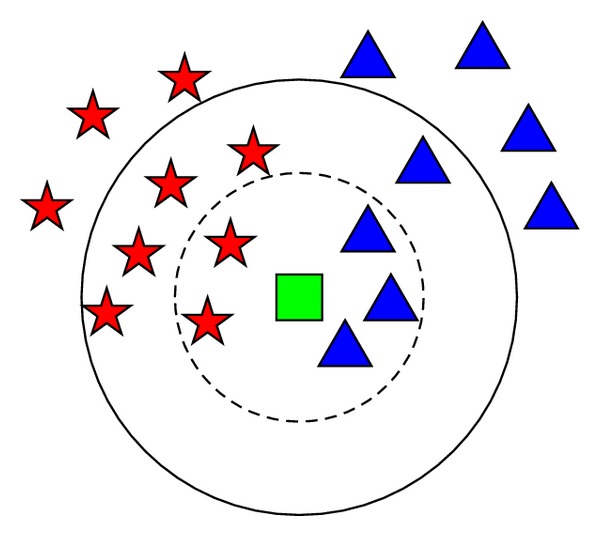
The principle diagram of the kNN classification algorithm.

Figure 2 is the principle diagram of the kNN classification algorithm.

As shown in Figure 2, the green square represents the sample to be classified. It needs to be classified into red five-point star or blue triangle. It is obvious that it is classified to blue triangle while *k* is set to 5, since the probability of classifying it into blue triangle is 60%, which is higher than that of classifying it to red five-point star (40%). While *k* is set to 10, the green square is classified into red five-point star, since the probability of classifying it into red five-point star is 60%, higher than the probability of classifying it into blue triangle (40%).

### 3.2. Vehicle Trajectory Pattern Matching

#### 3.2.1. Spatial-Pattern Matching

After spatial patterns learned from motion trajectories, motion patterns under normal conditions are obtained, which are called normal motion patterns since then. If a new trajectory is in line with one of normal motion patterns, it says that no anomaly occurs. Otherwise, it says that the vehicle is running in an abnormal state. That is, traffic anomaly occurs.

This paper adopts the kNN classification algorithm to test a new trajectory. For the new trajectory, its real-time motion tracepoints are recorded, denoted as *v*_*i*_  (*i* = 1,…, *n*). We use kNN to make prediction on these motion tracepoints. The detailed steps are as follows.

1. For each tracepoint *v*_*i*_, we compute its DTW distance to each trajectory in normal motion patterns and sort the trajectories according to the DTW distances in ascending order. Then, we obtain the nearest *t* trajectories of the tracepoint *v*_*i*_.
2. Find the corresponding trajectory patterns that the nearest *t* trajectories belong to. Assuming that there are *t*_*j*_  (0 ≤ *t*_*j*_ ≤ *t*) nearest neighbors that belong to the *j*th normal motion pattern (*j* = 1,…, *k*), the probability of the tracepoint *v*_*i*_ belonging to the *j*th normal motion pattern is *t*_*j*_/*t*.
3. Follow the same procedure to calculate its probability belonging to each normal motion pattern for each new tracepoint according to steps (1) and (2).

Based on the predictions of the tracepoints of the new trajectory, we can estimate whether the new trajectory changes motion patterns. If the new trajectory has an obvious shift, changing from one normal motion pattern to another, this indicates that the vehicle changed the driving lane during the running procedure, and it belongs to an abnormal behavior. With this approach, we achieve real-time abnormal traffic behavior recognition.

#### 3.2.2. Direction-Pattern Matching

A motion pattern has a certain direction. When the direction of a new trajectory is not the same as the one of the directions of normal motion patterns, traffic anomaly must occur. Using the direction of the central trajectory of each cluster as the direction of the corresponding motion pattern reduces the computational complexity of a trajectory testing. After pattern learned from motion trajectories, this paper uses the direction information of motion patterns to detect abnormal behavior of moving vehicles.

It first obtains a direction vector *c* to present the central trajectory of a motion pattern. When testing a tracepoint *v*_*i*_ reached, we assume that the direction of the first testing tracepoint is in accordance with the direction of its cluster central trajectory. For other tracepoints (i.e., *i* > 1), the direction discrimination of abnormal behavior detection is as follows:

(2) $$\begin{array}{c} \alpha =\text{arccos}\;\frac{b\cdot c}{\left|b\right|\left|c\right|}, \end{array}$$

where *α* is the angle between the testing trajectory and its cluster central trajectory and *b* is the direction vector from the tracepoint *i* − 1 to the next one *i* of the testing trajectory. When *α* ≤ 90°, the testing trajectory is running in accordance with the normal direction of the motion pattern. Otherwise, the testing trajectory is running backwards.

### 3.3. Mixed Pattern Matching-Based Abnormal Behavior Recognition

**Figure 3:**
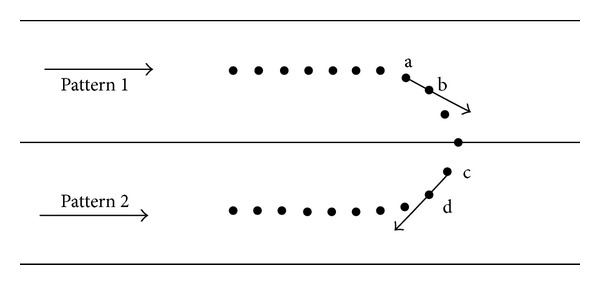
The principle diagram of mixed pattern matching-based abnormal behavior recognition.

The principle diagram of an individual vehicle abnormal behavior recognition based on mixed pattern matching is shown in Figure 3.

As shown in Figure 3, the direction of the spatial pattern 1 and 2 is from left to right. Black points in the figure represent testing tracepoints. We predict that the testing trajectory shifts from the spatial pattern 1 to the spatial pattern 2. Meanwhile, the direction change occurs. That is to say, the vehicle is running backwards in the spatial pattern 2. It can be seen easily that the angle between the direction from *a* to *b* and that of the central trajectory of the spatial pattern 1 is less than 90 degrees. Thus, the vehicle moves according to a normal direction at this time. However, the angle between the direction from *c* to *d* and that of the central trajectory of the spatial pattern 2 is more than 90 degrees. Therefore, the vehicle is moving in a reverse direction, which is an abnormal behavior.

The procedure of abnormal behavior recognition is as follows.

Input: motion patterns obtained by pattern learning.

1. If it is the first testing trajectory tracepoint, we use the kNN classification algorithm to classify which spatial pattern it belongs to and set its motion direction in accordance with the direction of the central trajectory of the spatial pattern.
2. When the *i*th trajectory tracepoint comes, we also use the kNN classification algorithm to classify which spatial pattern it belongs to. If the motion pattern between the *i*th tracepoint and the *i* − 1th tracepoint is not the same, the testing trajectory is changing the lane.
3. We compute the angle between the vector direction from the *i* − 1th testing tracepoint to the *i*th testing tracepoint and that of the central trajectory of the motion pattern that the *i*th test trajectory tracepoint belongs to. If this angel is more than 90 degrees, it indicates that the testing trajectory is running in a reverse direction.

## 4. Instance Analysis of Abnormal Behavior

This paper used traffic videos of actual traffic scene to verify the vehicle abnormal behavior recognition method. The traffic videos of urban bayonets and highways are selected as testing objects for verifying the recognition effect of vehicle abnormal behaviors. Program is implemented using Matlab 2010a, and the hardware configuration as follows: Pentium(R) Dual-Core CPU E5300@2.60 GHz, 4 G memory, Windows 7 operating system.

### 4.1. Lane-Changing Behavior Recognition

**Figure 4:**
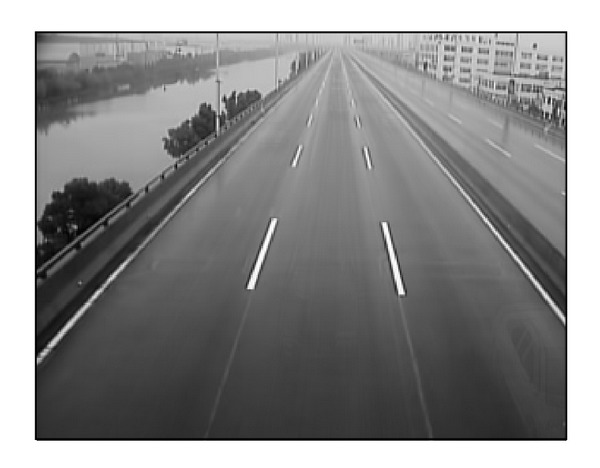
A source image showing a traffic video scene of a highway.

Figure 4 is a selected traffic scene of a highway.

**Figure 5:**
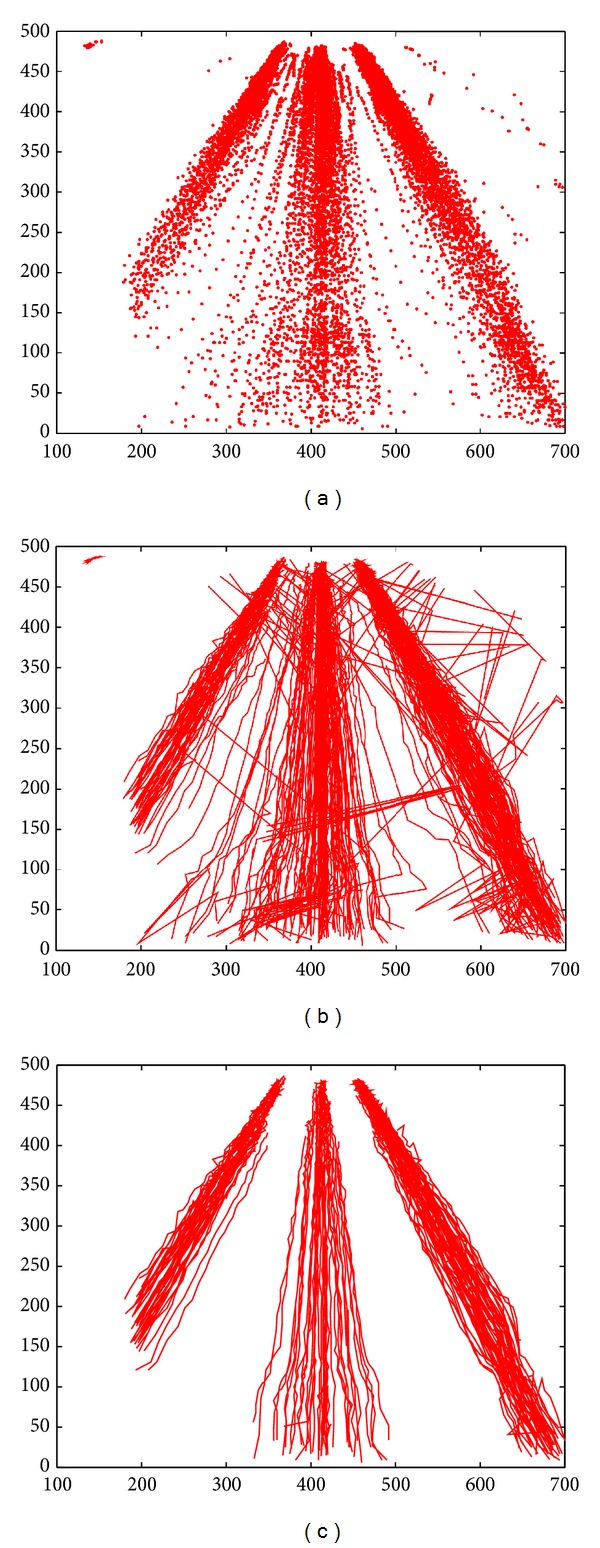
The extraction and preprocessing of vehicle trajectories of a highway road. (a) Tracepoints of vehicle motion trajectories; (b) trajectories after interpolation processing; (c) trajectories after smoothing and redundancy removing.

The tracepoints of moving vehicles by object tracking are shown in Figure 5(a).  Figure 5(b) is the image of motion trajectories after interpolation processing. From Figure 5(b), it can be clearly seen that the extracted trajectories have noises because of a variety of reasons. Under normal circumstances, motion trajectories of moving vehicles in the same lane should be in the same direction. After performing smooth processing and redundancy removing on Figure 5(b), the processed result is shown in Figure 5(c).

**Figure 6:**
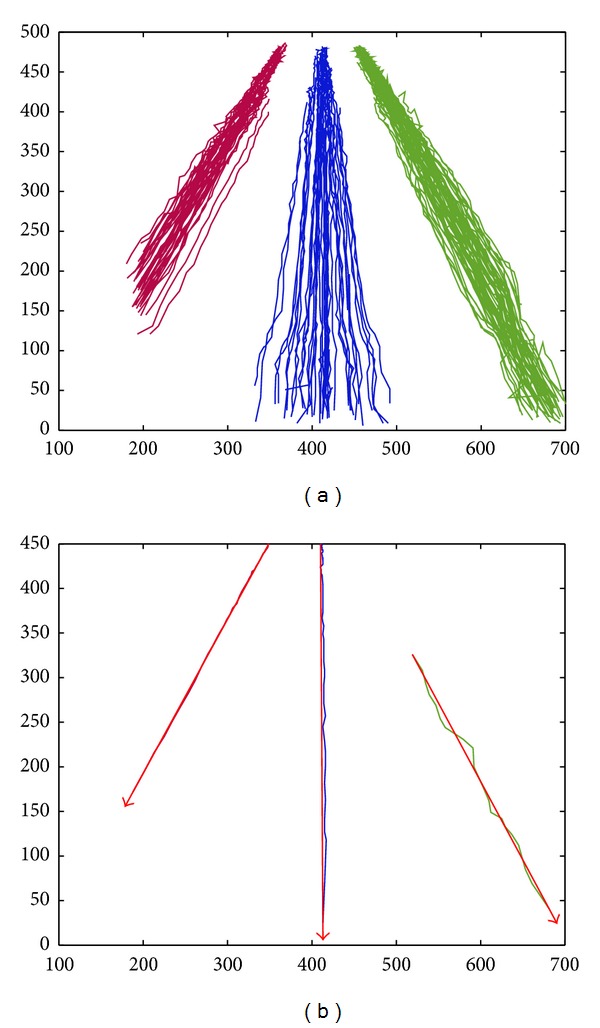
Pattern learning results of vehicle trajectories of a highway road. (a) The spatial patterns of trajectories. (b) The direction patterns of trajectories.

We perform clustering based on DTW and spectral clustering on trajectory sequences obtained after redundancy removal, and the results are shown in Figure 6.  Figure 6(a) shows the clustering result of the spatial patterns of trajectories. We can also see that the trajectory sequences are separated into three trajectory clusters via the DTW spatial distance and spectral clustering, which are distinguished with three different colors. The traffic scene of this experimental video has three running lanes. Under normal driving, there exist three spatial behavior patterns.

On the basis of three trajectory spatial patterns obtained by spectral clustering as shown in Figure 6(a), the direction patterns of trajectories can be obtained according to the method proposed in Section 2.4. The results are shown in Figure 6(b). It showed that there are three vehicle trajectory lines which are the closest to the trajectory centers of three clusters, respectively, and the directed arrows from a start point to an end point of these three trajectories represent the direction patterns of the trajectory clusters.

**Figure 7:**
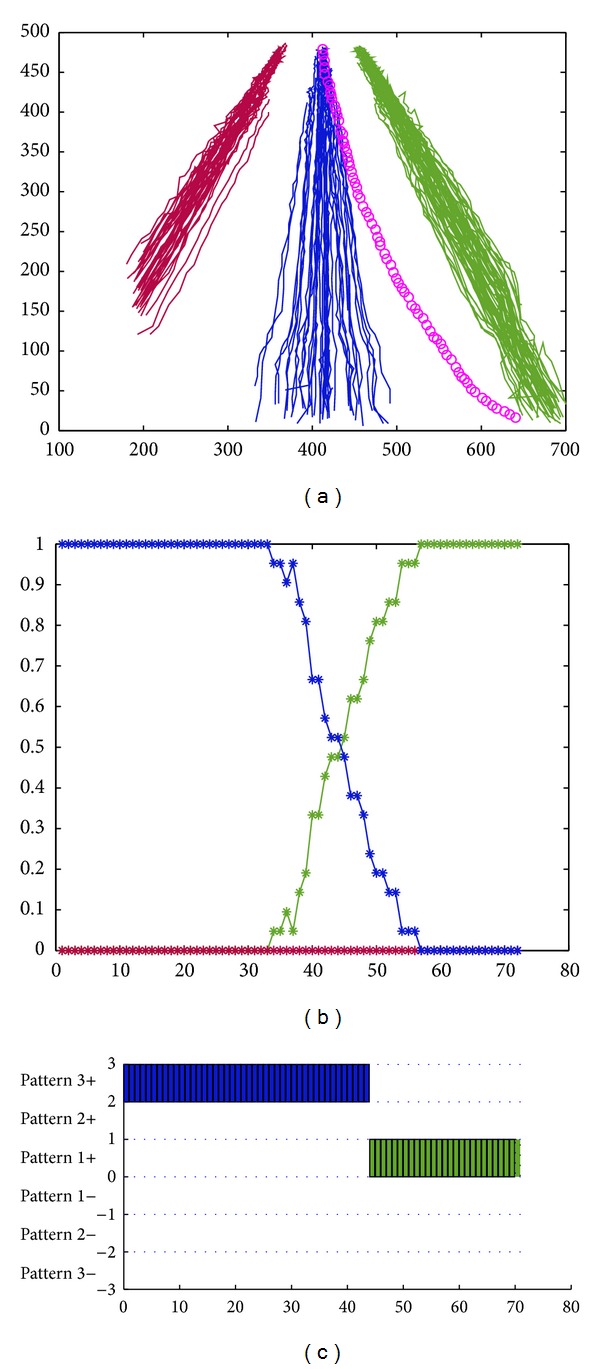
Changing lane behavior recognition: (a) an abnormal trajectory, (b) the matching results of the spatial patterns, and (c) the matching results of mixed patterns.

On highway, if a vehicle wants to change a lane, it should turn on signal in advance to call attention from the following car. But there always exists the abnormal lane-changing phenomenon, which easily results in traffic incidents. It is one of high-risking driving behaviors. In Figure 7(a), the motion trajectory marked with pink is a changing lane behavior. Figure 7(a) also shows the clustering results.  Figure 7(b) shows spatial-pattern matching results. For the testing trajectory, at the beginning it traveled in the middle motion pattern, that is, the motion pattern marked in blue color. Therefore, the probability belonging to the blue pattern is 1, and the probability belonging to the red pattern and that of the green pattern are 0. With its gradual shift, the vehicle shifted to the green motion pattern and finally ran on the green motion pattern.  Figure 7(b) shows that the probability of the blue motion pattern decreases gradually, downward to 0. The probability of the green motion pattern increases gradually, upward to 1. The probability of the red motion pattern always keeps as 0. Note that the sum of the probabilities belonging to the three patterns is 1. After integrating direction pattern matching with the basis of spatial pattern matching, the final abnormal recognition result is shown in Figure 7(c).

In Figure 7(c), we assigned a number to represent each pattern: 1 for the green pattern, 2 for the red pattern, and 3 for the blue pattern. We can see from Figure 7 that this vehicle shifted from pattern 3+ to pattern 1+. Note that the “+” after the pattern numbers represents the direction of the vehicle. Therefore, we can see that the changing lane abnormal behavior occurred.

### 4.2. Illegal Retrograde Recognition

**Figure 8:**
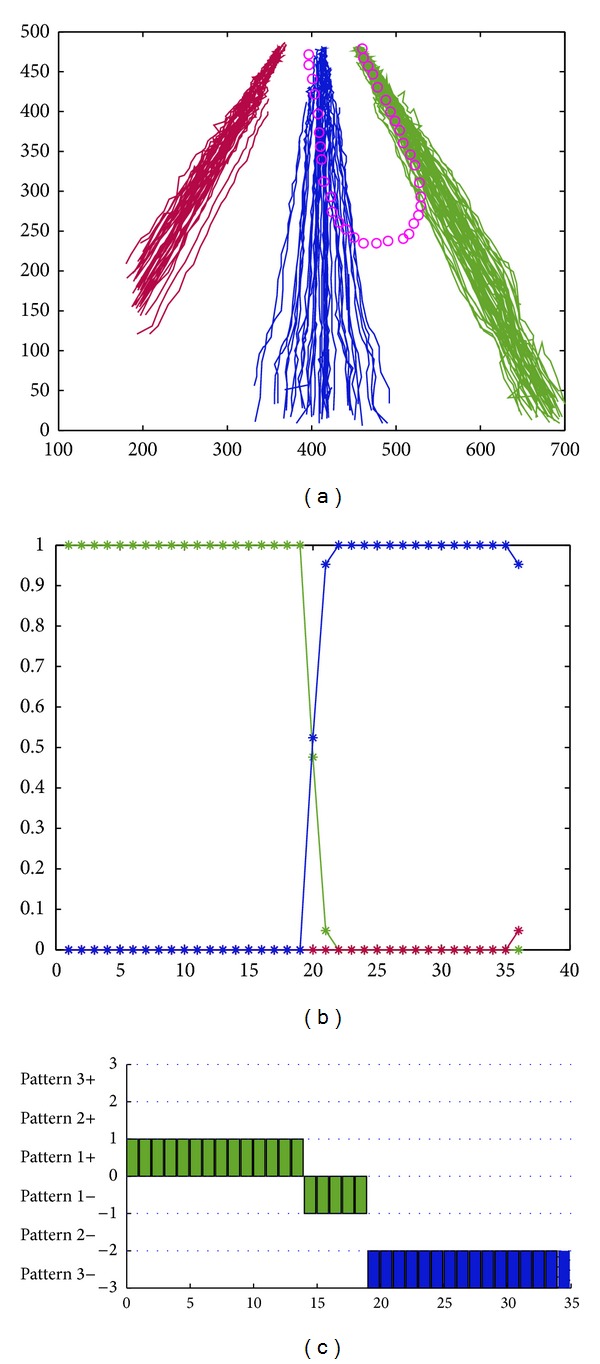
Illegal retrograde behavior recognition. (a) An abnormal trajectory. (b) The matching results of the spatial pattern. (c) The matching results of the mixed pattern.

We still use the above highway traffic scene. On highway, it sometimes appears risking behaviors like illegal retrograde. In Figure 8(a), the motion trajectory marked with pink is an abnormal illegal retrograde. This kind of abnormal behaviors can be detected using the above clustering approach.  Figure 8(b) shows the spatial pattern-matching result of the test trajectory. From the figure, we can see that the vehicle traveled in the right motion pattern at beginning, that is, the motion pattern marked in green. The probability belonging to the green pattern is 1, and the probability belonging to other patterns (red and blue) is 0. With the gradual shift of the vehicle, it shifted to the blue motion pattern. Meanwhile, its motion direction shifted from forward to backward gradually, and finally it ran in the blue motion pattern in a reverse direction. This has been reflected by Figure 8(b).  Figure 8(b) shows that the probability of the green motion pattern decreases gradually, downward to 0. The probability of the blue motion pattern increases gradually, upward to 1. The probability of the red motion pattern always keeps as 0. After integrating direction pattern matching with the basis of spatial pattern-matching result, the final abnormal recognition result is shown in Figure 8(c).

In Figure 8(c), this vehicle firstly shifted from pattern 1+ to pattern 1− and then from pattern 1− to pattern 3−. Note that the “−” after the pattern numbers represents the reversion direction. We can see that this vehicle completed the whole traveling procedure of retrograde. Combing the context information in the traffic scene, the illegal retrograde abnormal behavior is detected.

### 4.3. Guiding-Violation Behavior Recognition

**Figure 9:**
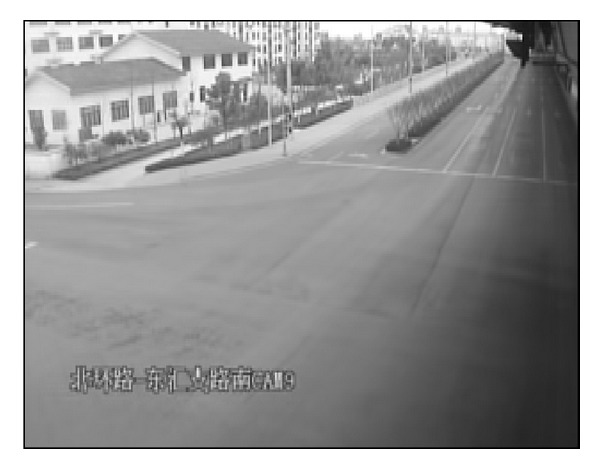
A source image showing a traffic video scene of an urban bayonet.

Figure 9 is a selected traffic scene of an urban bayonet.

**Figure 10:**
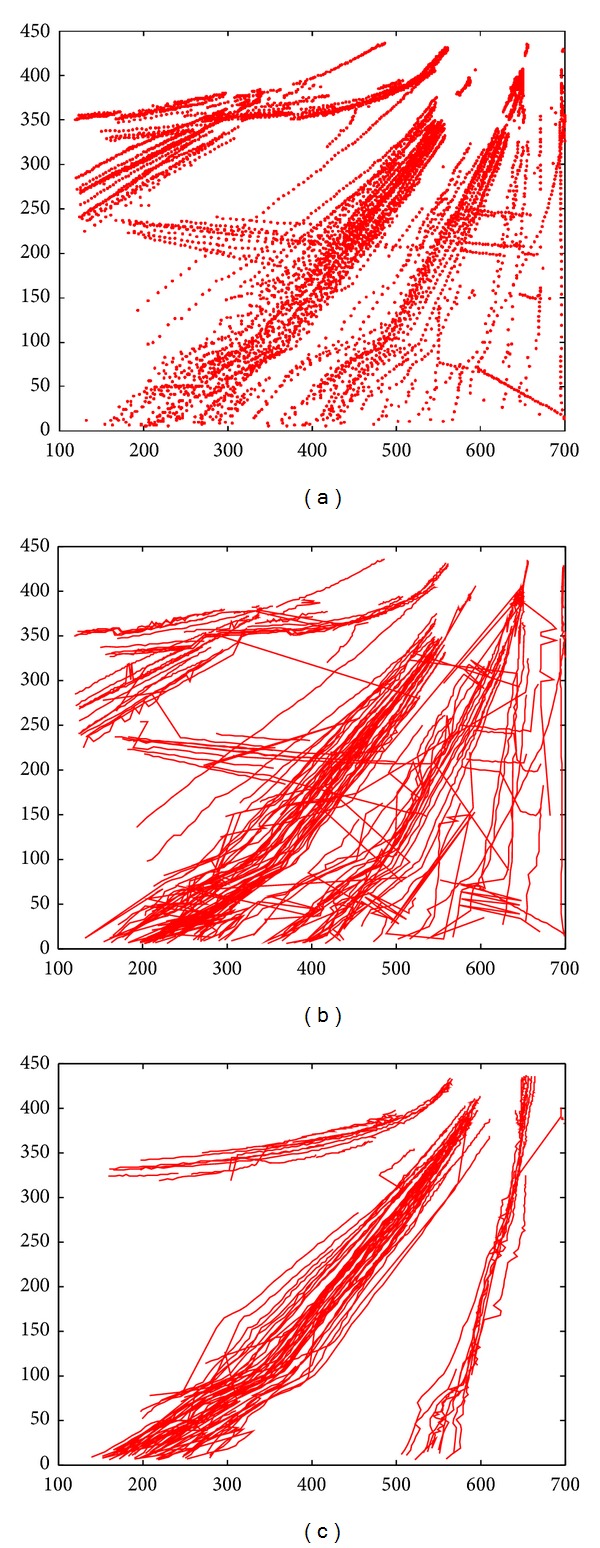
The extraction and preprocessing of vehicle trajectories of an urban bayonet: (a) tracepoints of vehicle motion trajectories, (b) trajectories after interpolation processing, and (c) trajectories after smoothing and redundancy removing.

The tracepoints of running vehicle trajectory sequences collected by a motion tracking algorithm are displayed in Figure 10(a). Because urban traffic is more complex, there exist more noises. After interpolation processing and redundancy removing, they are restored to original coordinates as shown in Figures 10(b) and 10(c).

**Figure 11:**
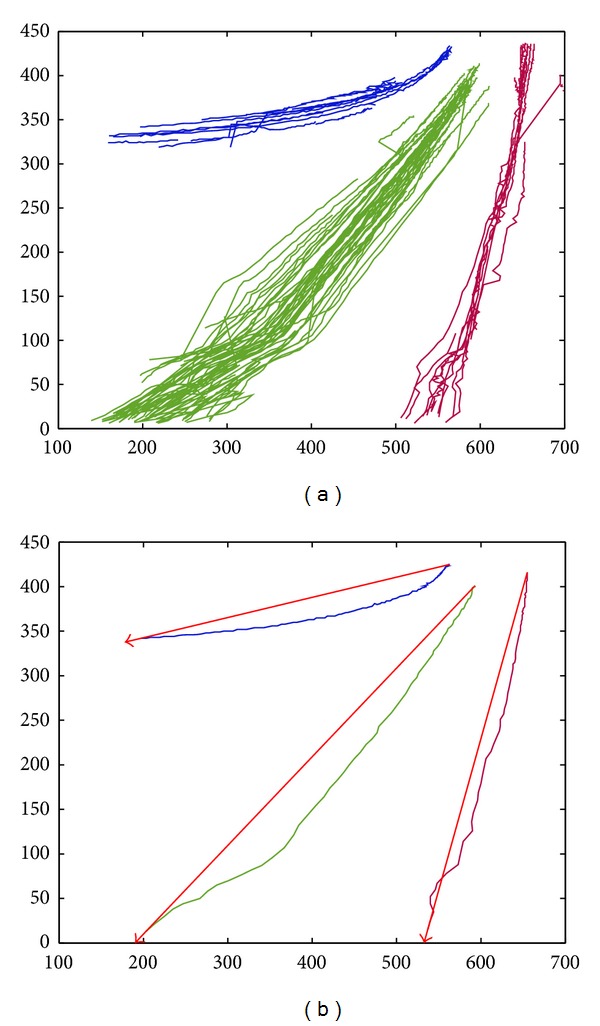
Pattern learning results of vehicle trajectories of an urban road. (a) The trajectory spatial patterns. (b) The trajectory direction patterns.

The clustering results of vehicle trajectories patterns in Figure 11 are obtained by the above clustering steps. We noticed that the clustering results are not four clusters. Main motion patterns are just three clusters. This indicates that vehicle traffic behavior patterns are affected by a variety of factors, rather than merely road settings. Again, we use different colors to mark different normal motion patterns. The experiments show that the obtained trajectory patterns using the clustering method proposed in this paper reflected the actual traffic situation.

**Figure 12:**
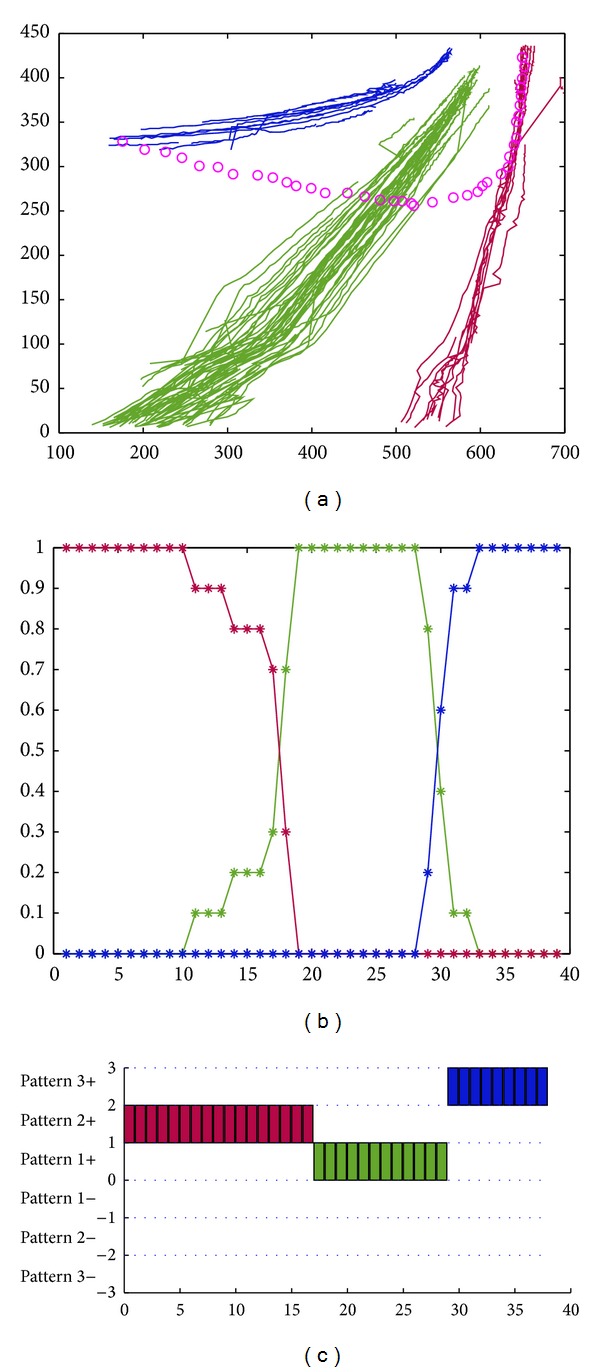
Guiding-violation behavior recognition. (a) An abnormal trajectory; (b) the matching results of spatial patterns; (c) the matching results of mixed patterns.

The anomaly recognition process is shown in Figure 12.

In Figure 12(a), the motion trajectory marked with pink is a common traffic guiding-violation behavior in an urban road. That is, the vehicle changed to another lane after it entered a guiding lane. We used the above clustering approach to recognize this kind of vehicle abnormal behaviors.  Figure 12(b) shows its spatial-pattern matching results. For the test trajectory, it traveled in the right motion pattern at beginning, that is, the motion pattern marked in red color. Thus, the probability of this trajectory belonging to the red pattern is 1, and the probability belonging to both the green pattern and the blue pattern is 0. With the gradual shift of the vehicle, after it shifted to the green motion pattern, it shifted to the left blue motion pattern. This has been reflected in Figure 12(b).  Figure 12(b) shows that the probability of the red motion pattern decreases gradually, downward to 0. The probability of the green motion pattern increases gradually, upward to 1. Then, the probability of the blue motion pattern increases gradually, upward to 1. Combining the direction pattern matching with the basis of the spatial pattern matching result, the final abnormal recognition result is shown in Figure 12(c). In this figure, we also present the patterns using numbers: 1 for the green pattern, 2 for the red pattern, and 3 for the blue pattern. This figure shows that this vehicle shifted from pattern 2+ to pattern 1+ first and then from pattern 1+ to pattern 3+. Therefore, we can see that this vehicle completed the whole travel procedure from driving straightly to turning right. Combing the context information in the traffic scene, the guiding-violation abnormal behavior is detected.

## 5. Conclusions

This paper proposed an effective method for traffic abnormal behavior recognition based on trajectory analyses. We first introduced the DTW distance to measure the distance between vehicle trajectories and put forward a trajectory pattern learning method based on DTW and spectral clustering. In order to extract the spatial-patterns and the direction-patterns of vehicle trajectories, this paper constructed a DTW distance matrix and determined the number of clusters automatically by the spectral clustering algorithm. After that, sample data points are clustered into different groups according to the features of trajectories. Then, this paper put forward a recognition method for vehicle abnormal behaviors based on mixed pattern matching, which integrated spatial patterns and direction patterns learned from trajectories. The experimental results showed that the proposed technical scheme can recognize main types of traffic abnormal behaviors effectively. Because traffic behaviors of moving vehicles are complex and diverse and have a strong randomness, how to recognize and analyze more complicated abnormal behaviors is an important issue needed to be further investigated while intelligent videos enter into industrial applications.
